# Supplementary material for: Yiai Fuzheng decoction inhibits triple-negative breast cancer by remodeling the immune microenvironment
Source: Front Immunol. 2025 Sep 30;16:1615631. doi: 10.3389/fimmu.2025.1615631 (PMC12518410; doi:10.3389/fimmu.2025.1615631)
Supplement: Supplementary file 2 [file DataSheet1.zip › Supplementary File 2/Treatment/C_vs_D/07_Pathway_Analysis/Pathway_Analysis/Sig_Pathways_ID/Fructose and mannose metabolism.pdf]

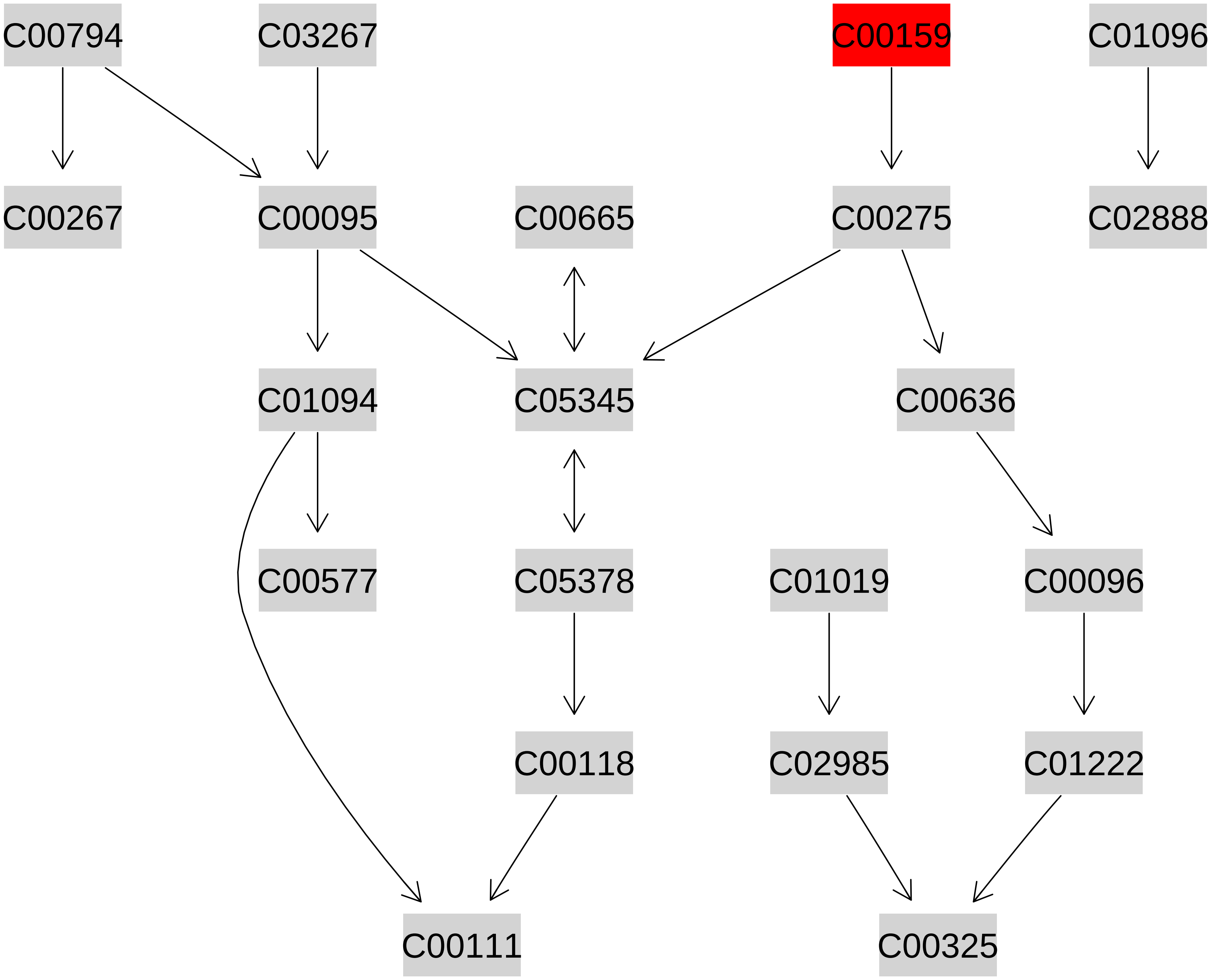
C00794
C03267
C00159
C01096
C00267
C00095
C00665
C00275
C02888
C01094
C05345
C00636
C00577
C05378
C01019
C00096
C00118
C02985
C01222
C00111
C00325
